# Analysis of heterogeneity and epistasis in physiological mixed populations by combined structural equation modelling and latent class analysis

**DOI:** 10.1186/1471-2156-9-43

**Published:** 2008-07-08

**Authors:** Mogens Fenger, Allan Linneberg, Thomas Werge, Torben Jørgensen

**Affiliations:** 1 Department of Clinical Biochemistry and Molecular Biology, University Hospital of Copenhagen, Denmark; 2 Research Centre for Prevention and Health, Copenhagen County, Glostrup University Hospital, Denmark; 3 Psychiatric Research Centre, Skt. Hans Hospital, Roskilde, Denmark

## Abstract

**Background:**

Biological systems are interacting, molecular networks in which genetic variation contributes to phenotypic heterogeneity. This heterogeneity is traditionally modelled as a dichotomous trait (e.g. affected vs. non-affected). This is far too simplistic considering the complexity and genetic variations of such networks.

**Methods:**

In this study on type 2 diabetes mellitus, heterogeneity was resolved in a latent class framework combined with structural equation modelling using phenotypic indicators of distinct physiological processes. We modelled the clinical condition "the metabolic syndrome", which is known to be a heterogeneous and polygenic condition with a clinical endpoint (type 2 diabetes mellitus). In the model presented here, genetic factors were not included and no genetic model is assumed except that genes operate in networks. The impact of stratification of the study population on genetic interaction was demonstrated by analysis of several genes previously associated with the metabolic syndrome and type 2 diabetes mellitus.

**Results:**

The analysis revealed the existence of 19 distinct subpopulations with a different propensity to develop diabetes mellitus within a large healthy study population. The allocation of subjects into subpopulations was highly accurate with an entropy measure of nearly 0.9. Although very few gene variants were directly associated with metabolic syndrome in the total study sample, almost one third of all possible epistatic interactions were highly significant. In particular, the number of interactions increased after stratifying the study population, suggesting that interactions are masked in heterogenous populations. In addition, the genetic variance increased by an average of 35-fold when analysed in the subpopulations.

**Conclusion:**

The major conclusions from this study are that the likelihood of detecting true association between genetic variants and complex traits increases tremendously when studied in physiological homogenous subpopulations and on inclusion of epistasis in the analysis, whereas epistasis (i.e. genetic networks) is ubiquitous and should be the basis in modelling any biological process.

## Background

Despite tremendous progress in our understanding of the human genome and the rapidly advancing ability to probe genetic variation, investigation of the human genome has yielded only modest insight into the pathogenesis of complex human traits such as glucose levels and insulin resistance. One possible explanation is inadequate modelling of complex polygenic interactions and phenotypic heterogeneity.

First, as far as a condition or disease is monogenic in the sense that a single gene variant is sufficient to precipitate a condition or disease, family-based studies are powerful in detecting causal genes or at least in narrowing down the location of the genes on the chromosomes [1]. However, most conditions and diseases of a major impact on public health, such as type 2 diabetes mellitus, are complex, with each factor presumably only contributing minimally to the trait in the general population [2].

Although it is acknowledged, that most common diseases are complex, the traditional Mendelian single-gene approach is still common (see [3] for a discussion). The implicit assumption is that a single gene, or a few major genes, determines the outcome of a condition, even for complex conditions. Concomitant gene-gene interaction or epistasis is often either ignored completely or deemed unimportant. However, epistasis is a physiological reality in all biological systems and is thus one of the most important features in complex conditions [4]. No gene is an island but exclusively exerts its function through interaction with other genes in integrated networks [5-8]. In fact, it can be argued that even apparently monogenic diseases are not truly monogenic, but rather can be considered as complex polygenic traits caused by mutations showing varying degrees of penetrance [9]. Ignoring the importance of genetic and non-genetic interactions is one of the main reasons for the rather meagre and contradictory results of genome association studies [10-13].

Second, genetic studies of complex diseases initially pursued the notion that the sole important phenotype to study was the conventionally defined diagnosis of the disease. This is somewhat peculiar given that most complex diseases are characterised by clinical heterogeneity, which would argue against a uniform etiological cause. As this incoherence is now widely acknowledged, genetic studies have been broadened to include quantitative trait loci (QTL). Although this strategy has been somewhat successful, it has rarely provided insight into the complex disorder itself. One reason for this is that, although dynamic, continuous traits are introduced, most genetic studies are still performed as case-control studies and thus dichotomise the trait or phenotype. This approach inevitably leads to loss of power [14]. Another issue is that study populations are rarely homogeneous, and although the study population is often stratified (e.g. by gender, age or ethnicity), this is frequently rather arbitrary in nature and may even be irrelevant to the process studied, hence reducing the power of the study.

Several methods have been developed to account for population heterogeneity and epistasis [3,15-32]. Newer advanced clustering methods originating from computer science have been introduced, including fractal analysis, genetic programming and non-linear dynamics [33-37]. These latter techniques incorporate gene-gene interactions and non-genetic variables to explain phenotypic variability. They have the weakness as most other methods, that they depend on genetic models and in particular on *a priori *defined penetrance functions of interactions. These methods are to various extents based on coalescence theory and they use of genetic data for classification mostly in hierarchy tree-like structures, which is rather unrealistic in natural systems [38]. An alternative is to model networks of interacting genes, gene products, and regulatory structures covering the entire genome [6-8,39], but even in mono-cellular organisms this is a daunting task and is far from being resolved. The hypothesis is that any observable phenotype of a complex biological system by necessity is determined by the complexity of its underlying biochemical organisation and the signalling network operating within it. It is the entire network behaviour and not a single, specific variable that determines the physiological outcome.

Here we adopt a "naive" approach, focussing on resolving physiological heterogeneity related to the metabolic syndrome and thus predisposition to type-2 diabetes in the general population using structural equation modelling (SEM) in a latent class analysis (LCA) framework. The assumption is that all physiological processes examined are coded for and regulated by genomic structures in an extensive cellular and inter-cellular network and are modified by non-genetic factors to varying extents. The population as a whole is considered to consist of a finite amount of physiologically distinct subpopulations defined by the state of the genetic network. The purpose of this analysis is not to define the disease, but rather to define subpopulations that are characterised by physiologically distinct metabolic entities or *states *that may or may not develop into type 2 diabetes mellitus, which is recognised to be a heterogeneous condition [40] with a large polygenic genetic component [41]. The second step in this study is an evaluation of the importance of resolving population heterogeneity in genetic studies by SEM-LCA analysis.

## Methods

### Population and variables

The population used is that of the Danish Inter 99 study, which comprises all 61,301 individuals born at 5-year intervals between 1939 and 1970 in 11 municipalities in south-western Copenhagen County. From the original population an age- and sex-stratified random sample of 13,016 individuals was contacted and invited to participate in the Inter 99 study of which 6,775 responded to the invitation to participate [42].

Fasting levels of glucose, insulin, C-peptide, lipids and cholesterol were analysed, and blood pressure and anthropometric measures such as height, weight, body mass index (BMI) and waist/hip circumference (WH) were obtained. In addition, an oral glucose tolerance test (OGTT) was performed in all individuals, and glucose, insulin and C-peptide were measured at 0, 30 and 120 min. BMI and WH are included as indices of obesity. Insulin resistance (HOMAres) and β-cell function (HOMAbeta) were calculated using fasting levels of insulin and glucose [43]. Basic variables for the study population are summarised in Table 1.

**Table 1:** Summary of the variables in the Inter 99 study used in the SEM-LCA analysis

	Mean (SD)	
	Women	Men

Number	3,345	3,169
Age (years)	45.8 (8.0)	46.6 (7.8)
BMI (kg/height2)	25.8 (5.1)	26.8 (4.0)
Waist-hip ratio (WH)	0.80 (0.06)	0.92 (0.06)
Systolic blood pressure	127 (18)	134 (17)
Diastolic blood pressure	80 (11)	85 (11)
Total cholesterol (mmol/L)	5.4 (1.1)	5.6 (1.1)
LDL (mmol/L)	3.4 (1.0)	3.7 (1.0)
VLDL (mmol/L)	0.53 (0.28)	0.67 (0.36)
Triglycerides (mmol/L)	1.15 (1.23)	1.54 (1.39)
C-peptide 0 min (pmol/L)	577 (256)	627 (301)
C-peptide 30 min (pmol/L)	1,954 (662)	2,054 (769)
C-peptide 120 min (pmol/L)	2,394 (967)	2,245 (1,078)
Insulin 0 min (pmol/L)	41 (28)	45 (31)
Insulin 30 min (pmol/L)	281 (166)	302 (201)
Insulin 120 min (pmol/L)	228 (203)	210 (229)
Glucose 0 min (mmol/L)	5.4 (1.0)	5.8 (1.2)
Glucose 30 min (mmol/L)	8.2 (1.8)	9.2 (1.9)
Glucose 120 min (mmol/L)	6.3 (2.0)	6.2 (2.5)
HOMAres	1.71 (1.61)	2.00 (1.69)
U-albumin/creatine ratio (mg/mmol)	8.9 (52.4)	8.4 (60.4)

All variables except glucose at 120 min and the U-albumin/creatinine ratio are significant different between genders (p < 0.001).

A total of 30 single nucleotide polymorphisms (SNPs) in 21 genes previously shown to be associated with the metabolic syndrome and diabetes mellitus were included in the genetic part of this study [see Additional file 1].

The study was approved by The Ethics Committee of Greater Copenhagen, Denmark.

### Modelling heterogeneity

Heterogeneity is modelled in a latent class framework combined with SEM. The modelling is performed by applying a finite mixture model or LCA [44,45]. The probability structure assumed for the variables in the LCA is of the form *f*(**y**|**z**) = Σ_*x*_π(**x**|**z**)*f*(**y**|**x,z**). We are modelling the probability density of observing **y **(= insulin levels) given a set of covariates **z **(= glucose, C-peptide, etc.). Here π(x|**z**) is the probability of having a certain set of values for the discrete latent variable (**x**, "liver") given an individual's observed covariates. π(x|**z**) is equivalent to the probability of belonging to a subpopulation and sums to 1 for each subject. At the population level, π(x|**z**) reflects the relative size of a subpopulation and sums to 1. The outcome of this modelling is a classification of the population sample in mutually exclusive subpopulations with significantly distinct physiological metabolic states differing in their propensity to evolve into a clinical endpoint, in this case diabetes mellitus. In this model, the dependent or indicator variable (insulin at time points during the OGTT) is modelled as a continuous normal distribution *f(y|x) *conditional on class membership in a perfect classification. A number of known physiological risk factors for the metabolic syndrome or type-2 diabetes were introduced as independent variables in the model (see Results section on Model building for details).

#### Model assumptions

All subjects are assumed to possess exactly the same basic genetic structures, but these vary in expression because of variability in the genome, including SNPs, deletions, insertions and copy number variations, and due to environmental factors. Furthermore, it is assumed that the population consists of a mixture of subpopulations, within which all variables are presumed to be normally distributed and only correlated through a latent variable. Variables may not be exactly normally distributed in a tissue or organism because of asynchrony of the dynamic processes in the cells [46], but the modelling approach used here is robust to minor deviations from normality. The hypothesis is that the difference in variables between subpopulations is defined by distinct genetic variations in the subpopulations. Genetic structures and variations are not modelled directly, but are embedded in the latent variable. The variance in measured variables included in the model reflects the genetic variability that we eventually want to elucidate. It should be stressed that no particular genetic model is assumed other than that all potential genes are part of a network common to all subjects. No assumptions are made for the distribution of traits in the basic study population as such. In particular, a normal distribution of traits in the general population is not required; in fact, a normal distribution in the basic study population may indicate a single physiologically homogeneous population, which is not expected for the metabolic process. In addition, if traits in the basic study population are actually normally distributed, the population may be truly homogenous and no physiological mixture of subpopulations would be present conditional on the trait of interest.

**Figure 1:**
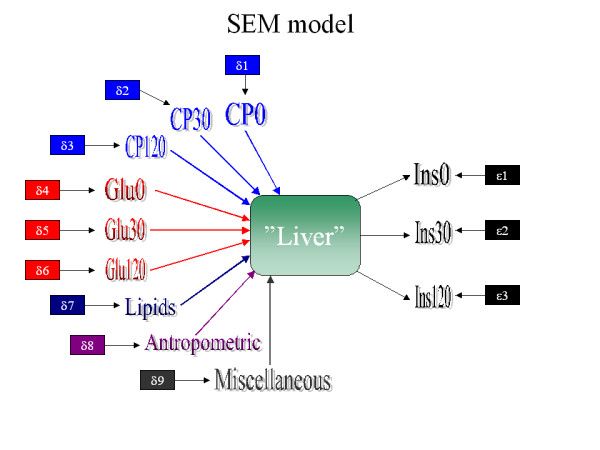
The figure illustrates the simplified model used in the present study. As explained in Methods liver symbolize a surrogate of processes in the tissues with focus on the insulin metabolism. C-peptide denotes the secretion of C-peptide and hence insulin from the pancreatic β-cell. The insulin on the right side of the liver indicates the amount of insulin actually reaching the general circulation influencing the metabolism in peripheral tissues. Most of the insulin execute its action in and is internalized by the liver. Glucose and lipids are both metabolised in the liver, but also in many other tissues, in addition to influencing insulin production and secretion in the pancreas. All these processes can of course be modelled, but at the moment it will impose severe computer challenges. Nevertheless, this highly simplistic model turns out to be very efficient in the latent class analysis (see text).

The structural model (SEM) of the metabolism of glucose and hence the metabolic syndrome is schematically shown in Figure 1. The metabolic syndrome is conceived as diminished glucose utilisation (uptake and processing of glucose) in peripheral tissues caused by increasingly inefficient action of insulin, i.e., insulin resistance evolves in the tissues. "Increasingly" should be conceived both as differences in insulin response between homogeneous subpopulations determined by the subpopulation genotype and as a modulation of insulin resistance by non-genetic factors. The pancreatic β-cell is the sole physiological source of insulin for which synthesis and secretion are influenced by numerous substances, including glucose. However, most of the insulin does not reach the general circulation, as it is metabolised in its first passage through the liver. Only 15–30% of the insulin secreted from the pancreas actually reaches the general circulation. The actual secretion by pancreatic β-cells can be estimated by measuring C-peptide, which is cleaved from proinsulin when insulin is secreted from the pancreas. C-peptide is not cleared by the liver and therefore reflects the β-cell activity. However, we are interested in the general metabolic state of the organism and therefore the biological activity of insulin (rather than in its production), and thus we define insulin as the principal indicator of this metabolic state. Nonetheless, the actual production of insulin is still an integrated circuit in the metabolic pathways, and C-peptide is therefore included into the model as a covariant (Figure 1). Several metabolites other than glucose influence the metabolic process that in turn also directly influences the secretion of insulin from pancreatic β-cells, but complete modelling of these pathways is complicated and computer- intensive and will be the subject of future studies. Nevertheless, the simple model used here is very efficient (see below), and therefore all latent variables are modelled in one complex variable, which we name "liver" as a surrogate for all tissues involved in glucose metabolism and insulin resistance, recognising the over-simplification this implicates. Note that no covariates are allowed to directly affect insulin, because, although correlated to insulin, they are not directly explanatory, but act through cellular processes.

It is presumed that no subject transitions between classes are possible. Only metabolic transitions from one level to another level of the metabolic status would be within a class, depending on the load of non-genetic factors and within the limits defined by the class-specific genotype, i.e., the non-genetic factors operate within the limits of a genetic framework that physiologically cannot be exceeded.

#### Data mining

SEM-LCA analysis is time-consuming and computer-intensive. Therefore, an exploratory ordinary linear regression analysis was performed as a mean of data-mining to select variables most correlated to insulin. The selected variables were used in the initial building of the model. After the basic model was developed, the remaining variables correlated to insulin or previously shown to be correlated to the metabolic syndrome were successively entered into the analysis and were retained in model if they increased its goodness of fit.

### Genetic analyses

The entire study population was genotyped using the SNPlex technique (ABI Biosystems) or Taqman chemistry (ABI Biosystems). SNPs with unresolved ambiguities (range 3.2 – 8.5%) were excluded from the analysis. The minor allele frequencies ranged from 0.08% to 48.83%. Hardy-Weinberg equilibrium (HWE) was analysed using the exact method of Wigginton et al. [47]. The R-script provided by Wigginton et al. was slightly modified to an S-Plus script, including calculations of confidence intervals. Two-gene linkage disequilibria (LD) were calculated for all combinations of genes included in the study. The methods of Weir [48] for calculating di-, tri – and quadrigenic disequilibria were adopted.

### Heritability and epistasis

The heritability of traits was calculated for each SNP [49]. For genes with multiple SNPs, i.e., angiotensinogen (AGT), interleukin 6 (IL6), adrenergic receptor β-2 (ARβ2) (each genotyped for two SNPs), and hepatic nuclear factor 4α (HNF4α; seven SNPs), heritability was calculated for each SNP separately. Two-gene physiological epistasis was calculated for all combinations of SNPs for each trait by variance decomposition as previously described [50-52].

### Statistics and programmes

The exploratory data-mining procedure to select variables included in the model, and comparisons of variables in the classes were performed using the SPSS package v.15.0.

Structural analysis is carried out using Mplus v.4.0, which can perform LCA and SEM in a single operation [53]. The corrected Bayesian information criterion (BIC) was used as the decision criterion for the best models generated. Algorithms for calculation of HWE [47], two-gene disequilibria [48], and epistasis [50-52] were all programmed in S-Plus v.7.0 (Insightfull).

## Results

### Variables in the model

An exploratory linear regression analysis was performed for the entire population and for the two subpopulations stratified by gender. The latter is justified, as the variables related to the metabolic syndrome and diabetes are known to differ between genders [41] (see below). The purpose of this analysis was to identify the variables with most potential influence on the model indicators, i.e., insulin at 0, 30, and 120 min (Figure 1). Analysis revealed that variables related to the metabolic syndrome and diabetes were also significantly correlated to insulin levels, albeit to different extents (data not shown). Insulin, C-peptide, BMI, and very low density lipoprotein (VLDL) were included in all the regressions, and age was included in all but one regression. Insulin at 0 min was only regressed as a covariate for 30- and 120-min OGTT results. Similarly, insulin at 30 min was only regressed as a covariate for 120-min OGTT results. Somewhat surprisingly, fasting glucose (at time 0) only entered two of the nine regressions, indicating that basic glucose levels do not influence the dynamics of insulin levels during a glucose challenge.

C-peptide at 0 min did enter the regression at 30 min, but not at 120 min. This may reflect two processes in C-peptide (and insulin) metabolism: secretion and catabolism. In the initial phase of the glucose challenge, insulin secretion (as measured by C-peptide levels) proceeded, but in the later phase secretion decreased, perhaps even below fasting levels, and the decrease in C-peptide and insulin levels mainly reflect the removal and degradation of the peptides. This scenario may pertain to what is perceived as normal metabolism, but collapses as insulin resistance and β-cell dysfunction progress.

In summary, insulin as an indicator and C-peptide and glucose as co-variates were used to build the basic model.

### Structural equation modelling and latent class analysis

Combined SEM and LCA was initiated by including insulin at the three time points as indicators without any co-variates. The number of classes was increased to the point at which the adjusted BIC value did not significantly change. Next, glucose and C-peptide were included separately and in all combinations as co-variates. Although glucose and C-peptide at 0 min were only significantly associated with the indicators in a fraction of the regressions, they were indispensable in the model, as assessed by the adjusted BIC criterion (Table 2, Stage 1; AIC and unadjusted BIC are included in the table for comparison of goodness-of-fit statistics). This results in a basic model with 19 classes or subpopulations for both men and women.

**Table 2:** Latent class model for women and men

**Indicators:**	Insulin levels at 0, 30 and 120 min after an oral glucose tolerance test (OGTT).								
**Covariates in all models:**	Glucose and C-peptide levels at 0, 30 and 120 min after OGTT.								
	Stage 1			Stage 2					**Final model**
**Women**									
Number of classes	17	18	19	18	18	18	18	18	19
Additional covariates				BMI	WH	TG	HDLC	Chol.	BCage^c^
df ^a^	551	583	615	746	746	746	746	746	1,188
AIC	85,626	81,531	8,.472	8,.330	81,487	81,473	81,411	81,490	80,962
BIC	86,322	82,870	8,.886	82,769	82,925	82,911	82,849	82,928	82,694
Sample-Size Adjusted BIC	85,947	82,148	8,.124	81,994	82,150	82,136	82,074	82,152	81,759
Entropy	0.876	0.868	0.859	0.872	0.862	0.873	0.872	0.871	0.879
Chi-tests:^b^									
AIC		<E-5	<E-5	<E-5	<3*E-4	<E-5	<E-5	<6*E-4	<E-5
BIC		<E-5	-	<E-5	-	-	<0.24	-	<0.33
BICadj		<E-5	<0.027	<E-5	-	<0.75	<E-5	-	<E-5
**Men**									
df	551	583	615	746	746	746	746	746	1,188
AIC	83,973	83,840	83,792	83,643	83,792	83,726	83,688	83,722	83,226
BIC	85,237	85,181	85,210	85,084	85,233	85,166	85,129	85,162	84,962
Sample-Size Adjusted BIC	84,556	84,459	84,447	84,309	84,457	84,391	84,354	84,387	84,028
Entropy	0.886	0.883	0.882	0.888	0.881	0.881	0.884	0.884	0.898
Chi-tests:									
AIC		<E-5	<E-5	<E-5	<E-5	<E-5	<E-5	<E-5	<E-5
BIC		<E-5	-	<E-5	-	<0.65	<E-5	<0.37	<0.39
BICadj		<E-5	<0.51	<E-5	1.00	<E-5	<E-5	<E-5	<E-5

^a^df, degrees of freedom.

^b^Chi-square tests. The models were compared with the immediately preceding model. The models in Stage 2 were compared to the best model in Stage 1 and so on.

^c^BCage, BMI, cholesterol and age.

The latent class variable was regressed on all covariates (see Figure 1B). All models with higher numbers of classes have lesser goodness-of-fit statistics and are omitted form the table.

The basic model was extended by including all the remaining variables separately as co-variates. The number of classes was varied for each variable included to ensure that an optimum was reached. The modelling results for the variables significantly entering the model are shown in Table 2, Stage 2. Note that the optimum number of classes was reduced from 19 to 18 at this stage of model building, irrespective of the variable entering the model. The co-variates BMI, WH, triglycerides, high density lipoprotein (HDL), and cholesterol were then evaluated in all combinations. In addition, variables that did not singularly improved the model fit were re-tested in combination with any additional co-variate entering the model. This process resulted in a final 19-class classification (Table 2, Final model), in which BMI, cholesterol, and age entered the model as covariates in addition to the basic covariates glucose and C-peptide. The building process was consistent in the sense that when the variable leading to the greatest improvement in the goodness-of-fit was included in the model, it remained in the model regardless which covariate was added in the next step.

Analysis of the final model was repeated five times using different programme seeds and increasing numbers of iterations to ensure that a global maximum in the fit was obtained, although it is difficult to prove that the solution of the model fit was in fact global and not local. However, exactly the same parameters and goodness-of-fit statistics emerged for all analyses and subject allocation in the classes was the same in all runs. Calculating the final model consisting of 19 classes and 12 variables took approximately 16 h on a 4-Ghz 1-GB-RAM stand-alone machine. The final model included insulin at all time points as indicators, glucose and C-peptide at all time points as covariates, and BMI, cholesterol and age. The number of classes was 19 for both men and women and exactly the same covariates are included in the model for both sexes. When the number of classes was increased to 20 or more, the model deteriorated (data not shown). The entropy measure [53] was almost 0.9, indicating a very high rate of correct classification of individuals in classes.

### Phenotype differences between classes

Classes are ordered according to an increase in insulin resistance defined by the HOMAres index [43]. The size of the classes and the fraction of frank diabetics identified is shown in Table 3. All the variables included in the study were evaluated for significant differences between gender and between the 19 classes within each gender. Except for glucose at 120 min, all variables were significantly different between the genders (data not shown). Within each gender there was a high proportion of significant differences between classes, even when the comparisons were corrected for multiple testing [see Additional file 2]. The differences in proportions were particularly true for insulin and C-peptide, whereas the number of differences was somewhat lower for the other variables in the model, in particular for cholesterol.

**Table 3:** Size of classes and distribution of frank diabetes type 2 (DMT2) cases in the classes

	**Women**			**Men**		
Class	Class size	No DMT2	% DMT2	Class size	No DMT2	% DMT2
1	140	2	1.43%	112	1	0.89%
2	93	0	0.00%	148	2	1.35%
3	163	0	0.00%	133	1	0.75%
4	243	0	0.00%	106	0	0.00%
5	233	0	0.00%	241	4	1.66%
6	197	3	1.52%	222	1	0.45%
7	118	0	0.00%	138	25	18.12%
8	129	8	6.20%	148	0	0.00%
9	173	0	0.00%	160	0	0.00%
10	259	0	0.00%	128	3	2.34%
11	176	3	1.70%	155	2	1.29%
12	141	0	0.00%	88	0	0.00%
13	95	0	0.00%	195	36	18.46%
14	90	3	3.33%	146	16	10.96%
15	95	5	5.26%	135	2	1.48%
16	102	1	0.98%	116	3	2.59%
17	103	23	22.33%	38	0	0.00%
18	58	20	34.48%	179	39	21.79%
19	71	11	15.49%	120	11	9.17%

	2,679	79	2.95%	2,708	146	5.39%

It was anticipated that the fraction of frank diabetic cases in the classes would increase with increasing class number (i.e., with increasing insulin resistance). However, this was clearly not the case, in particular not for men. A possible reason is that the HOMAres index is an inaccurate measure of insulin resistance, as it only includes glucose levels at 0 min, whereas insulin does not enter in the algorithm. Rather, the dynamics of glucose, insulin and C-peptide in the OGTT have to be implemented in analysing the metabolic status. Glucose levels in female classes 8 and 16–19 at 120 min and in class 18 at 0 min did indicate impaired glucose tolerance (IGT) according to the WHO criteria [40]. These five classes included 463 cases, or 17.3% of the entire female population. Except for class 16, all of these IGT-classes had increasing insulin levels at 120 min in the OGTT. In addition, classes 1 and 6 also showed increasing insulin levels at 120 min, albeit at a modest level.

Nevertheless, the latter classes did show increasing insulin levels concomitant with normalised glucose levels at 120 min, i.e., full compensation of insulin resistance was obtained for these two classes (which would not be detected by the HOMAres index). The combined insulin resistance classes (defined as IGT or increasing levels of insulin at 120 min after the glucose challenge) amount to a total of 895 females, or 33.4% of the female study population. Only five classes, including 782 or 29.2% of the females, showed decreasing C-peptide levels after 120 min, indicating increased secretion of C-peptide (and hence insulin) in the remaining 70% of the female population. On an individual level, approximately one-third of the women had increased secretion of C-peptide (Table 4). This discrepancy between individual and class-average gradients indicates that a substantial fraction of the classified women had not reached their final dynamic state within the class. This difference is much less pronounced in men (Table 4). The situation is reversed for insulin gradients: 70–75% of the individuals exhibited a positive gradient, but far fewer were included in classes with positive average gradients. It should, however, be remembered, that insulin levels are the result of a complex process of secretion and catabolism involving interaction with the insulin receptor (particularly in the liver), whereas C-peptide mainly reflects the secretory process. Taken together, the results presented in Table 3 and 4 indicate that the WHO criteria for defining insulin resistance and the concept of insulin-glucose homeostasis need to be re-evaluated. In addition, the model presented here may be too simplistic.

**Table 4:** Dynamic metabolic status and gradients for insulin and C-peptide

			Gradients^c^		
	Status	(WHO)		Insulin	C-peptide
**Women**					
Population	NGT^a^	82.5%	Negative^d^	30.2%	65.3%
	IGT^b^	17.5%	Positive	69.8%	34.7%
Classes	NGT	82.7%	Negative	70.4%	29.2%
	IGT	17.3%	Positive	29.6%	70.8%
**Men**					
Population	NGT	72.8%	Negative	24.9%	55.2%
	IGT	27.2%	Positive	75.1%	44.8%
Classes	NGT	81.1%	Negative	81.1%	41.0%
	IGT	18.9%	Positive	18.9%	59.0%

^a^NGT, normal glucose tolerance; IGT, impaired glucose tolerance. According to the WHO criteria (ref. 40)

^b^IGT, defined as glucose at 0 minutes above 6.1 mmol/l and/or glucose at 120 minutes above 7.8 mmol/l

^c^Gradients calculated as the differnece between the 120 min and 30 min levels.

^d^Negative: negative gradients; Positive: positive gradients

### Genetic analysis

#### HWE

A total of 30 SNPs in 21 genes were examined in the population sample. Among these, only the SNP termed HNF4α-3 [see Additional file 1] deviated from HWE after correction for multiple testing in both the female and male subpopulations (Table 5). However, after stratification by SEM-LCA, this SNP was in HWE in approximately 75% of the classes.

**Table 5:** Hardy-Weinberg equilibria

			Number	Genotypes			Frequence		
Gene				AA^b^	Aa	aa	Allele A	Allele a	P-value^a^
**Gender**	HNF4a -3	Male	2,908	1,996	629	283	0.80	0.20	8.8E-65
	HNF4a -3	Female	3,052	2,111	667	274	0.80	0.20	5.2E-59

		Class							
**Men**	HNF4a -3	1	138	96	25	17	0.79	0.21	5.0E-07
	HNF4a -3	2	221	140	51	30	0.75	0.25	4.3E-08
	HNF4a -3	3	208	142	41	25	0.78	0.22	1.1E-08
	HNF4a -3	10	131	86	27	18	0.76	0.24	2.2E-06
	HNF4a -3	11	85	65	12	8	0.84	0.16	8.7E-05
	HNF4a -3	12	142	98	29	15	0.79	0.21	2.5E-05
	HNF4a -3	15	125	95	13	17	0.81	0.19	3.6E-11
	HNF4a -3	17	185	126	41	18	0.79	0.21	2.8E-05

**Women**	HNF4a -3	3	182	134	30	18	0.82	0.18	6.2E-08
	HNF4a -3	7	90	73	10	7	0.87	0.13	4.5E-05

^a^Only genes with p-value below the Bonferoni corrected p-value are shown

^b^The most abundant allele is arbitrary labelled A

#### Linkage disequilibrium

Two-SNP LD analysis [48] was performed within genders before and after LCA (data not shown). There were 435 possible combinations for each gender, and 8,265 combinations for each gender after LCA stratification into 19 classes. LD analysis (i.e., statistical association between SNPs [48]) was performed in two steps: LD between SNPs within the same gene (HNF4α, AGT, IL6, and Arβ2), and LD between SNPs that are not in physical linkage, i.e., on different chromosomes or far apart on the same chromosome [see Additional file 1].

The total fraction of all possible two-SNP combinations in LD after correction for multiple tests decreased from 4.3 % before stratification to 1.3% and 1.4% for women and men, respectively, after classification. The physical unlinked SNP pairs increased from 21% before stratification to more than 56% after classification. Although these fractions of SNP combinations in LD seem small, they indicate that stratification into subpopulations increases the number of physically unlinked genes in LD, suggesting a joint influence on class membership, i.e., epistasis (see below). Concomitantly, the number and fraction of spurious LDs due to admixture decreased significantly. Most LDs were di-allelic, but a few tri- and quadri-allelic LDs were present, indicating stringent genotype-combinations influencing the traits.

Interestingly, all the HNF4α polymorphisms were in mutual LD except for HNF4α-6 (data not shown) in both genders before stratification, but most LDs were lost after stratification, except for SNPs HNF4α-2 and HNF4α-3. In contrast, only a few physically unlinked SNPs were in LD before stratification, but this number was 15-fold greater after stratification. Only the LDs in the non-stratified gender population between HNF4α-2/HNF4α-3 and Arβ2-1/ARβ2-2 were recovered in almost all classes. Thus, most of the LDs detected in the non-stratified population disappeared after classification, whereas several new LDs emerged after classification. This finding may be interpreted as hidden epistasis in non-stratified populations, as the SEM-LCA was based solely on physiological variables (no genes were included in the SEM-LCA analysis). This suggests that the non-stratified but admixed population exposes two problems: the creation of false LDs and masking of true LDs [17].

### Heritability

When the population sample was stratified according to gender, none of the SNPs showed even a marginal effect on any of the physiological variables (Table 6). When genders were stratified into LCA classes, a few SNPs in a few classes showed a significant and Bonferroni-corrected influence on a limited number of physiological variables, mainly urinary-albumin and urinary-creatinine.

**Table 6:** Heritability of the traits shown in Table T1

			Heritability					
	Class	Trait	Gene	Number^a^	Additive	Total	F-test	p-value
	Gender	None^b^						
**Women**	5	HOMAbeta	IL6-2	223	0.01	0.10	48.83	<1E-16
	9	Triglycerides	AP2β	163	0.15	0.54	20.25	<1E-16
	17	U-albumin	AGRP	100	0.31	0.33	15.06	2E-6
	17	U-alb/crea	AGRP	100	0.32	0.34	15.17	2E-6
	19	Ins30	PPAR	71	0.08	0.47	17.45	1E-6
**Men**	5	U-alb/crea	HNF4a-1	236	0.02	0.08	13.52	3E-6
	8	Triglycerides	AGT-1	141	0.11	0.25	17.28	<1E-16
	13	Triglycerides	ARβ3	190	0.01	0.10	23.35	<1E-16
	15	U-alb/crea	HNF4a-1	129	0.04	0.13	14.37	2E-6
	15	U-albumin	HNF4a-1	129	0.05	0.13	14.15	3E-6
	16	U-albumin	HNF4a-1	113	0.16	0.35	17.96	<1E-16
	16	Triglycerides	HNF4a-2	112	0.13	0.29	14.76	2E-6
	17	U-albumin	PGC1	37	0.21	0.96	19.72	2E-6
	19	U-albumin	AGT-1	119	0.12	0.25	29.41	<1E-16
	19	U-alb/crea	AGT-2	119	0.11	0.24	28.81	<1E-16

^a^Number of individuals included in the test

^b^Number of heritabilities detected for any trait

However, these were variables *a priori *not suspected to be causative in the metabolic syndrome and were not included in the final SEM-LCA model. For a nominal 5% level of significance, approximately 5% of all heritabilities were significant. This is, as expected, by pure chance and remarkably non-significant, considering that the genes were selected because several studies have shown that they influence various physiological variables related to the metabolic syndrome and type 2 diabetes mellitus [see Additional file 1].

### Epistasis

#### Pre-LCA analysis

To examine epistatic effects on the physiological variables that characterise the metabolic syndrom and type 2 diabetes, logistic regression analysis was performed on both genders prior to LCA stratification for all two-SNP combinations. The variance components of the analyses that were significant after correction for multiple testing (including the summary variables HOMAres and HOMAbeta) are shown in Table T3 [see Additional file 3]. In men, 180 two-SNP interactions did not influence any physiological variable, whereas 85 two-SNP interactions influenced all variables.

The numbers for women were 208 and 70, respectively. Combined for women and men, 165 two-SNP interactions did not influence any traits in either gender group, and 40 two-SNP interactions influenced all variables in both genders, which seems to suggest the existence of gender-specific interactions. Between 87.6% and 92.3% of the possible two-SNP combinations between different genes were involved in epistatic interactions termed *Real epistasis *in Table T3 [see Additional file 3] for both genders, in which the epistatic variance accounted for almost half of the genetic variance.

The single-gene multilocus genotypes HNF4α4, ARβ2, AGT and IL6 were explanatory of genetic variances even if single-locus SNPs were only explanatory in a few sporadic cases (Table 6). Nearly half of the genetic variance was accounted for by epistasis; the remaining genetic variance could be explained by two-gene additive and dominance variance.

Interestingly, all the functional SNPs (coding amino acids) were always included in the haplotypes of the HNF4α gene. This was also true for the AGT and ARβ2 genes. In the case of IL6, the two promotor SNPs were included for all the physiological variables examined, suggesting that they are both necessary in regulating IL6 expression.

#### Post-LCA analysis

The total number of possible two-SNP epistatic effects after classification into 19 subpopulations was 7,269 in women and 7,268 in men, or 87.9% of all possible interactions. Two-gene combinations in which one or both genes was monomorphic were excluded, as no interaction could be calculated. Combinations in which one or both genes was not in HWE were also excluded, as this was a prerequisite for the variance decomposition [52]. Of the two-SNP combinations included close to one-quarter significantly influenced at least one physiological variable in at least one gender-specific class after correction for multiple testing. *On average*, almost 10% of the phenotypic variances were accounted for by separate two-gene interactions across all physiological variables and all classes. The total genetic variance ranged from 0% to 66.8%, and the epistatic variance ranged from 0% to almost 40% of phenotypic variance.

The most striking results were that: (1) a few classes did not show any interactions at all for any physiological variable; and (2) stratification into subpopulations increased the genetic variance tremendously. The average total genetic variance increased by 30- to 35-fold compared to the non-stratified study population (Table 7). The smallest increase was more than 10-fold and the largest increase was more than 100-fold. This indicates that destructuring of the physiologically mixed population into more homogeneous subpopulations revealed masked genetic effects beyond simple two-gene effects. The epistatic effect amounted to approximately 50% on average of the total genetic variance.

**Table 7:** Summary of average of two-gene genetic variance (mean) as the fraction of total phenotypic variance stratified into subpopulations by LCA.

The significance level for inclusion of cases corrected for multiple comparisons is 6.1E-06						
	**Class**		**Average genetic variance**			
			Minimum	Average	Maximum	Fraction
**Women**	Total variance		29	97	270	
		LCA/non-LCA^a^	*104*	*346*	*965*	
	Additive variance		9	29	78	29.6%
		LCA/non-LCA	*117*	*388*	*1,102*	
	Dominant variance		6	19	50	19.3%
		LCA/non-LCA	*104*	*332*	*864*	
	Epistasis		14	50	153	51.1%
		LCA/non-LCA	*105*	*378*	*1,143*	
**Number of interactions**		2,188				
**Fraction of possible**		26.5%				
**Number of interactions/trait**		156				
**Men**	Total variance		39	97	430	
		LCA/non-LCA	*118*	*299*	*1,357*	
	Additive variance		9	29	118	30.0%
		LCA/non-LCA	*97*	*296*	*1,226*	
	Dominant variance		7	17	57	18.5%
		LCA/non-LCA	*102*	*270*	*857*	
	Epistasis		19	50	257	51.5%
		LCA/non-LCA	*124*	*329*	*1727*	
**Number of interactions**		2,042				
**Fraction of possible**		24,7%				
**Number of interactions/trait**		146				

^a^Increase in average genetic variance in the classes relative to the genetic variance before stratification in to classes by LCA.

Analysis of epistasis was performed using corrected significance levels corresponding to the 30 SNPs studied. However, as the number of SNPs will be increased in future studies, the adjusted significance levels will dramatically decrease, which poses the question as to whether truly epistatic effects will still be detected. When the epistasis analysis was corrected for multiple testing corresponding to analysis of 30 SNPs (as performed above), the original number of nominally significant two-SNP interactions was reduced to 70%. Surprisingly, further correction for multiple testing corresponding to analysis in a 20-million-SNP scenario – the assumed number of SNPs in the human genome [54] – only reduced the number of significant interactions to 60%. (Table 8). This decrease was mainly due to a loss of significant interactions detected in the non-stratified population. Remarkably, the number of interactions not detected in the non-stratified population but detected after stratification was rather high when the significance level for 30 SNPs was used (22.3%, Table 8), which increased to almost one-third when the significance level for the 20-million-SNP scenario was used. This is due to the concomitant decrease in the significance level on excluding interactions due to the assumption of HWE in the analysis of epistasis [52]. Taken together, the non-stratified populations were prone to falsely excluding significant epistasis. In contrast, the false discovery rate for non-stratified populations was very low (Table 8). It is of course expected that many more and probably new interactions will be detected when the number of genotyped SNPs is increased, including interactions with the SNPs included in the present study and interactions between SNPs hitherto not known to be associated with glucose metabolism or diabetes. In particular, it is expected that interactions with other genes or SNPs will be detected for the SNPs that did not show any interactions in the present study (TCF1 and HNF4α-6). TCF1 and HNF4α-6 were included in the present study because they have been shown to be associated with the metabolic syndrome and diabetes [see Additional file 1], and hence must be part of an interacting network (provided that the detected associations are not false; see the discussion below).

**Table 8:** Detected interactions in genders before and after SEM -LCA.

	Significance level			
	0.0017		2.5 E-9	
1 Detected in both gender and LCA	245	56.3%	196	45.1%
2 Not detected in either gender or LCA	61	14.0%	68	15.6%
3 Detected in both LCA but not in any gender	97	22.3%	133	30.6%
4 Detected only in gender	2	0.5%	2	0.5%
5 Detected in one LCA and in gender	23	5.3%	23	5.3%
6 Detected in one LCA but not in gender	7	1.6%	13	3.0%

Full agreement between both gender and LCA (1+2)	306	70.3%	264	60.7%
Detected in one or both LCA but not in gender (3+6)	104	23.9%	146	33.6%
Actual number of possible interactions	378			
SNPs with no interactions (equivalent for both scenarios): TCF1		HNF4α-6		

Number of interactions between the 30 SNPs included in this study at two Bonferoni corrected significance levels corresponding to a 30 SNP study (0.0017) and to a future 20-million-SNP scenario (2.5 E-9). Percentage indicates the fraction of possible number of interactions for 30 SNPs (i.e., 435)

Some classes did not show any two-gene interactions at all. This is summarised in Table 9 using the significance level for a 20-million-SNP study for the three dynamic components in the model (glucose, insulin, and C-peptide). One class for each gender (class 18 in women and class 17 in men) did not show any interactions at all for any genes. Several other classes did not show any interactions, particularly for insulin at 0 and 120 min. If only these traits were investigated, it could be falsely concluded that none of the genes are involved in the metabolic process in these classes. However, it is assumed that all subjects possess the same genetic network, but in these particular classes none of the genes, or rather their polymorphisms, influenced the variance of the metabolic process defined by the traits, suggesting that hitherto undetected polymorphic genes define the physiological state in these classes.

**Table 9:** Genes and classes with no interactions (20 million SNP significance level)

	Women	Men
Genes with no interactions in any classes and any traits:	TCF1, HNF4α-6	TCF1, HNF4α-6, MCHR1

Classes with no interactions at 0 minutes:		
Insulin	13, 14, 16, 17, *18*	4, 12, 16, *17*,19
C-peptide	13, 16, *18*, 19	4, *17*
Glucose	18	17
Classes with no interactions at 30 minutes:		
Insulin	7, *18*	17
C-peptide	18	17
Glucose	18	17
Classes with no interactions at 120 minutes:		
Insulin	7, 13, 16, *18*, 19	12, *17*, 19
C-peptide	18	17
Glucose	*18*, 19	7, *17*

## Discussion

This study used LCA to stratify a random population sample into physiologically homogeneous subpopulations with respect to the metabolic *state *of the participants by defining the metabolic process in a multivariate SEM. The most important conclusion emerging from this study is that LCA-SEM very efficiently defines metabolic substates and allows detection of epistasis and genetic variance. In contrast, analysis of single-gene polymorphism has very limited power to detect QTL. Only a small fraction of the single gene-variants (or SNPs) showed any influence on the physiological variables examined in this study (Table 6), even though the SNPs were previously implicated in the metabolic syndrome [see Additional file 1]. In addition, most of the (few) detected heritabilities concern variables reflecting kidney status that are not considered causative in the development of insulin resistance and are most likely spurious associations. Low replication rates in single-gene association studies have been reported in a recent survey of association studies [10]. A major reason for the low replication rate is that variations in genes affecting polygenic traits or conditions are likely to have a low marginal impact on the trait that is therefore difficult to detect, particularly in small samples. The concept of polygenic traits implies that single-gene effects depend on other genes, and consequently may not be detected in single-gene analysis except in the "lucky" instances for which the genetic "background" is the "right" one (see e.g. [5,55] for additional real-data examples). This does not indicate that gene variants with no detectable marginal effects in this study are not important.

They are still part of the genetic-metabolic network. Rather, the low replication rate reflects the challenge of single-gene studies in complex conditions and stresses that epistatic and network analysis needs to be the main framework when studying polygenic traits in homogenous populations or subpopulations. This conclusion is supported by a the recently published genome wide association study for 7 major diseases, including 2,000 cases each and 3,000 controls [11]. Only slightly more than a handful of SNPs or genes related to diabetes were detected, of which several had been detected in previous studies. The somewhat disappointing result of that study is, however, very important, as it may mark the limit of what can be obtained by traditional genetic approaches, at least in outbred populations. These issues are well recognised, but the computer power and software needed for analysis of epistasis in genome wide association studies are generally not available at present.

It is commonly argued that statistically significant genetic association can be falsely inferred by subdividing a study population. However, the low replication rate in single-gene association studies [10], could be interpreted as a consequence of comparing global subdivided populations: the differences between different populations are the consequence of the specific full-genome genotype that has evolved in each particular population. Therefore, caution should be observed when concluding that studies that cannot be confirmed in other populations, are "sporadic" and therefore should be dismissed. On the contrary, it may be that these non-replicated studies disclose real associations. This scenario will extend to the further stratification of a particular population based on physiological models, as applied in this study, as most or all natural stratified populations are not genetically homogeneous. System biological approaches [56] including network models will be needed to elucidate the true nature of the genes and their interactions.

In a network model it is not implicitly assumed that the various stages of the metabolic disease process evolve as a linear process. Rather, several routes in the network with different impacts on the metabolic process are possible and are in fact the only plausible interpretation of real biological systems [39]. This notion is supported in the present study by the non-linear increase in diabetic cases in the ordered classification presented in Table 2. Although simple in concept, network models are extremely complicated, not just because of the number of genes involved, but also because of the complex regulations of genes by non-coding regions of the genome, and not least because of the interactions and interdependence of cellular functions in multicellular organisms.

In the present study, maximum information on the metabolic process was collected for the entire study population, including a number of dynamic physiological variables, whereas crude clinical endpoints were excluded as being too simplistic and prone to losing essential information [14]. Using this approach, we found that C-peptide and glucose describe the metabolic process in the model to a large extent, as reflected by the high entropy measure (Table 2). One of the most surprising findings in the SEM-LCA analysis is that VLDL, triglycerides, HDL and blood pressure did not enter into the model, despite contrary findings in the initial, univariate regression analysis and even though decreased HDL and increased triglyceride and blood pressure are generally accepted characteristics of the metabolic syndrome [40,41]. In addition, WH circumference is not a covariate in the model, although this is a measure of visceral fat, which is thought to be of major importance in developing insulin resistance.

Instead, BMI entered as a general measure of fat content. One probable reason for the discrepancies is that previous studies have been performed on selected populations (i.e., diabetics) whereas our approach has been to consider the metabolic *state *(rather than the metabolic *syndrome*). Thus, we consider it to be a strength that our population study targets a "normal" population, which in fact contain (at least) 18% insulin-resistant subjects and 4.2% with undiscovered diabetes mellitus. One interpretation of these results is that the variables excluded (most notably triglycerides and HDL) are in fact not causative, but inaccurate proxies for the actual underlying causative agents, such as free fatty acids [57], leptin [58], and other biochemical and hormonal substances involved in metabolic regulation of the variables excluded.

The complexity of the network is indicated by the differences in gene variants that characterise the physiological variables in the classes. First, MCHR1 only contributes to phenotypic variance in one class for women (class 5), and not at all for men [see Additional file 4]. Second, several gene variants contribute to the variance in only a few classes. And third, there is a notable difference between women and men in the classes in which genes exclusively contribute to the variance. That is not to say that the gene variants not influencing phenotypic variance in a class are not part of the network.

Rather, in a particular class, the influence of a gene variant is overridden or neutralised by other interactions in the metabolic network to a point that is undetectable by the variables included in the model, or the genes are monomorphic in the class. It should be remembered that the model is a whole-body model: genes may be expressed in different tissues participating in different cellular networks, as well as expressing the epistatic effect *across *tissues.

All the genes (or SNPs) included in this study (except perhaps TCF1 and HNF4α-6) seem to be part of a genetic network or of sub-networks, as they are involved in epistatic interactions related to the metabolism of glucose and insulin, although the network or subnetworks cannot be exactly defined at the moment. TCF1 and HNF4α-6 did not show interaction with any of the SNPs included in this study. This, however, does not exclude the possibility that these transcription factors are embedded in a network. In the case of the HNF4α-6 SNP, six other SNPs in the HNF4α gene do show extensive epistatic action, so the gene is included in the network even if the HNF4α-6 SNP is functionally neutral. As for the TCF1 SNP examined, one obvious explanation for the lack of interaction is that the SNP is functionally neutral. Another reason may be that TCF1 is the sole representative of a subnetwork of the general network related to glucose and insulin metabolism and no interaction *per se *would be detectable. Metabolic networks most probably follows a small-world scale-free distribution of interactions [59,60], implying that between-gene correlation rapidly declines with inter-gene distance in the network [61,62]. Then, even if TCF1 is embedded in a subnetwork including other genes, it may be too distant in the subnetwork for any interactions to be detectable. If this is the case, it would be very difficult or even impossible to detect higher-order interactions by simply extending the variance decomposition as carried out here for two-gene interactions to higher-order interactions (apart from the problem of dimensionality rapidly reducing the power of any study). The solution to this problem is not simple and may include – among other approaches – incorporation of network theory and applications, extensive genotyping, and experimental confirmation of chemical and physiological interactions. Of course, a gene or a SNP may erroneously be found to be associated with a trait or condition for several reasons (e.g., admixture), but this does not seem to be the case for TCF1, as mutations in this gene are the cause of one form of maturity onset of diabetes in the young, MODY3 [63].

A strength of our approach is that allelic, genetic and trait heterogeneity, genetic admixtures, and phenocopies do not present an obstacle, because these entities are implicit in the SEM-LCA modelling framework. Allelic and genetic heterogeneity and genetic admixture (see, e.g., [64,65] for a review of the latter) are incorporated in the network model and are modelled by the latent variable. This includes genetic factors, as well as the interactions of genes and their products in the cell and regulatory structures in the genome. Trait heterogeneity is resolved by LCA. Phenocopies are basically the phenotypes of interest not explained by the factors or variables chosen for inclusion in a study. In the SEM-LCA context, phenocopies have no meaning, because the phenotype in question is defined in a common structure defined for all variants of the phenotype by all genetic and environmental factors.

The major difference between the approach adopted here and most of the study designs generally implemented (see papers cited in the Background) is that no genetic model is assumed other than that the genetic network shared by all participants is the basic structure of all biological processes. This does not mean that gene variability cannot or should not be included in the modelling. On the contrary, the ultimate purpose is to define the genetic network or networks, and genetic factors should be included in the modelling when they are solidly identified. This calls for various physiological, biochemical and cellular studies to eventually define the exact role of a gene and the nature of its regulation, which most probably is beyond the reach of classical associations studies. In recent years, several new approaches have been presented to elucidate complex biological structures [33-37,66-73].

These dynamic data mining and machine learning procedures include neuronal networks, genetic and evolutionary programming, and genetic algorithms, among others. These techniques are exciting, but are still in their infancy for complex biological systems, and have to be developed further.

## Conclusion

Any subject in a population will belong to different subpopulations, depending on the physiological process being studied, which is simply the consequence of independent segregating chromosomes and random mating. The extent of this gene-mixing process is of course modified by chromosomal linkage of genes, recombination, mutation (of any kind), migration, and selection, and to what extent random mating is in fact random. However, for every new generation the chromosomes during meiosis can be picked in 2^23 ^ways, meaning that the combinations in two merged meiotic cells run into the billions, far exceeding the human population. Of course, the variability is far from that number, but even in small networks the number of combinations is still daunting [39]. However, it has been shown, at least in unicellular organisms, that molecular networks are highly modular, so that most genes only participate in few circumscribed networks [38]. This seems to be a general biological principle. If it holds true for multicellular organisms, the complexity may be manageable, in particular if homogeneous physiological entities are defined as in the approach presented here. Then it should be possible to model networks beyond two-gene interactions, including non-coding regulatory structures [74].

## Authors' contributions

MF developed the SEM-LCA approach, carried out all the modelling and computations, and wrote the paper. TW participated in developing ideas and formulated parts of the paper. AL and TJ established the study population and provided all data.

All authors read and approved the manuscript.

## Supplementary Material

Additional File 1 **Genes previously shown to be involved in the metabolism of glucose and insulin. Included are 21 genes with a total of 30 single nucleotide polymorphisms**. The table lists the single nucleotide polymorphisms (SNPs) included in the study of heritability, linkage disequilibrium, and epistasis. Click here for file

Additional File 2 **Comparisons of traits between the 19 classes**. The table shows the number of significant differences of a trait between all two-class comparisons. Click here for file

Additional File 3 **Summary of two-gene genetic variance as the fraction of total phenotypic variance in gender before stratification into subpopulations by SEM-LCA**. The number of significant two-gene interactions and the variance decomposition for each variable examined in the study are summarized before stratification of each gender by SEM-LCA. Click here for file

Additional File 4 **Genes characterizing classes**. The table summarize genes involved in epistasis of insulin, C-peptide or glucose in a limited number of classes. In particular, the MCHR1 gene or SNP is only involve in epistasis in one class in women and none in men. Click here for file
