# Supplementary material for: Analysis of heterogeneity and epistasis in physiological mixed populations by combined structural equation modelling and latent class analysis
Source: BMC Genet. 2008 Jul 8;9:43. doi: 10.1186/1471-2156-9-43 (PMC2483291; doi:10.1186/1471-2156-9-43)
Supplement: Additional File 1 — Genes previously shown to be involved in the metabolism of glucose and insulin. Included are 21 genes with a total of 30 single nucleotide polymorphisms. The table lists the single nucleotide polymorphisms (SNPs) included in the study of heritability, linkage disequilibrium, and epistasis. [file 1471-2156-9-43-S1.pdf]

Tabel T1 Genes previously shown to be involved in the metabolism of glucose and insulin. Included are 21 genes with a total of 30 single nucleotide polymorphisms.

Gene	Symbol	Chromosome	Accession number	T-start[a]	Polymorphism	Reference
5,10-methylenetetrahydrofolate reductase	MTHFR	1p36.22	rs1801133		Ala222Val	[1]
Interleukin 6 receptor[b]	IL6R	1q21.3	rs8192284		Asp358Ala	[2]
Angiotensinogen[b]	AGT-1	1q42.2	rs4762	7509	Thr207Met	
	AGT-2		rs699	7692	Met268Thr	
Calpain-10	CAPN	2q37.3	rs3792267	Intron3	SNP44 G/A	[3,4]
Peroxisome proliferative activated receptor	PPARγ	3p25.2	rs1801282		Pro12Ala	[5]
Adiponectin	APM1	3q27	rs17300539	-11391	G/Apromoter	[6]
Peroxisome proliferative activated receptor, gamma, coactivator 1α	PGC1α	4p15.2	rs8192678		Gly482Ser	[7,8]
Adrenergic receptor β-2	ARβ2-1	5q32	rs1042713	264	Gly16Arg	[9]
	(ARβ2-2		rs1042714	297	Glu27Gln	[9]
Transcription factor AP-2 beta	AP2β	6p12.3	rs3798519	Intron1	A/C	
Interleukin 6	IL6-1	7p15.3	rs1800796	-597	promoter	[10]
	IL6-2		rs1800797	-572	promoter	[10,11]

Adrenergic receptor β-3	ARβ3	8p12	rs4994		Trp64Arg	[11]
Adrenergic receptor β-1	ARβ1	10q25.3	rs1801253		Gly389Arg	[12]
Potassium inwardly-rectifying channel, subfamily J, member 11	KIR6.2	11p15.1	rs5219		Glu23Lys	[13]
Apolipoprotein A-V	ApoAV	11q23.3	rs662799	-1140	promoter	[14]
Transcription factor 1 (Hepatic nuclear factor 1α)	TCF1	12q24.31			Arg583Gln	[15]
Agouti related protein	AGRP	16q22.1	rs5030980		Ala67Thr	[16]
Melanocortin 4 receptor	MC4R	18q21.32			Leu251Ile[d]	
G protein-coupled receptor 40[c]	GPR40	19q13.2	rs2301151		Arg211His	[17]
Glycogen synthase[c]	GYS1	19q13.33		-588	promoter	[18]
Hepatic nuclear factor 4α	HNF4α-1	20q13.12	rs1884614	-49.405	5'-upstream	[19]
	HNF4α-2		rs2425637	-5.875p	promoter	[19]
	HNF4α-3		rs2425640	-1.887	promoter	
	HNF4α-4		rs1885088	9.116	Intron 3	[19]
	HNF4α-5		rs1800961	12.410	Thr130Ile	[20,21]
	HNF4α-6			22.734	Val255Met	[20,22,23]
	HNF4α-7		rs3818247	27.556	3-UTR	[20]
Melanin-concentrating hormone receptor	MCHR1	22q13.2	rs133072		Asn32Asp	[24]

[a]Position from transcription site, only indicted were necessary to mark the distance between the polymorphisms.
[b]Distance between genes are 76 Mb
[c]Distance between genes are 14 Mb
[d]T.Hasnsen,,personal communication, Steno Diabetes Center, Gentofte, Denmark

Reference List

1. Linneberg A, Husemoen LL, Nielsen NH, Madsen F, Frolund L, Johansen N: **Screening for allergic respiratory disease in the general population with the ADVIA Centaur Allergy Screen Assay.** *Allergy* 2006, **61:** 344-348.

2. Hamid YH, Urhammer SA, Jensen DP, Glumer C, Borch-Johnsen K, Jorgensen T *et al.*: **Variation in the interleukin-6 receptor gene associates with type 2 diabetes in Danish whites.** *Diabetes* 2004, **53:** 3342-3345.

3. Fullerton SM, Bartoszewicz A, Ybazeta G, Horikawa Y, Bell GI, Kidd KK *et al.*: **Geographic and haplotype structure of candidate type 2 diabetes susceptibility variants at the calpain-10 locus.** *Am J Hum Genet* 2002, **70:** 1096-1106.

4. Horikawa Y, Oda N, Cox NJ, Li X, Orho-Melander M, Hara M *et al.*: **Genetic variation in the gene encoding calpain-10 is associated with type 2 diabetes mellitus [In Process Citation].** *Nat Genet* 2000, **26:** 163-175.

5. Ek J, Urhammer SA, Sorensen TI, Andersen T, Auwerx J, Pedersen O: **Homozygosity of the Pro12Ala variant of the peroxisome proliferation- activated receptor-gamma2 (PPAR-gamma2): divergent modulating effects on body mass index in obese and lean Caucasian men.** *Diabetologia* 1999, **42:** 892-895.

6. Vasseur F, Helbecque N, Dina C, Lobbens S, Delannoy V, Gaget S *et al.*: **Single-nucleotide polymorphism haplotypes in the both proximal promoter and exon 3 of the APM1 gene modulate adipocyte-secreted adiponectin hormone levels and contribute to the genetic risk for type 2 diabetes in French Caucasians.** *Hum Mol Genet* 2002, **11:** 2607-2614.

7. Andersen G, Wegner L, Jensen DP, Glumer C, Tarnow L, Drivsholm T *et al.*: **PGC-1alpha Gly482Ser polymorphism associates with hypertension among Danish whites.** *Hypertension* 2005, **45:** 565-570.

8. Ambye L, Rasmussen S, Fenger M, Jorgensen T, Borch-Johnsen K, Madsbad S *et al.*: **Studies of the Gly482Ser polymorphism of the peroxisome proliferator-activated receptor gamma coactivator 1alpha (PGC-1alpha) gene in Danish subjects with the metabolic syndrome.** *Diabetes Res Clin Pract* 2005, **67:** 175-179.

9. Ishiyama-Shigemoto S, Yamada K, Yuan X, Ichikawa F, Nonaka K: **Association of polymorphisms in the beta2-adrenergic receptor gene with obesity, hypertriglyceridaemia, and diabetes mellitus.** *Diabetologia* 1999, **42:** 98-101.

10. Hamid YH, Rose CS, Urhammer SA, Glumer C, Nolsoe R, Kristiansen OP *et al.*: **Variations of the interleukin-6 promoter are associated with features of the metabolic syndrome in Caucasian Danes.** *Diabetologia* 2005, **48:** 251-260.

11. Azuma N, Yoshimasa Y, Nishimura H, Yamamoto Y, Masuzaki H, Suga J *et al.*: **The significance of the Trp 64 Arg mutation of the beta3-adrenergic receptor gene in impaired glucose tolerance, non-insulin-dependent diabetes mellitus, and insulin resistance in Japanese subjects.** *Metabolism* 1998, **47:** 456-460.

12. Karlsson J, Lind L, Hallberg P, Michaelsson K, Kurland L, Kahan T *et al.*: **Beta1-adrenergic receptor gene polymorphisms and response to beta1-adrenergic receptor blockade in patients with essential hypertension.** *Clin Cardiol* 2004, **27:** 347-350.

13. Nielsen EM, Hansen L, Carstensen B, Echwald SM, Drivsholm T, Glumer C *et al.*: **The E23K variant of Kir6.2 associates with impaired post-OGTT serum insulin response and increased risk of type 2 diabetes.** *Diabetes* 2003, **52:** 573-577.

14. Pennacchio LA, Olivier M, Hubacek JA, Cohen JC, Cox DR, Fruchart JC *et al.*: **An apolipoprotein influencing triglycerides in humans and mice revealed by comparative sequencing.** *Science* 2001, **294:** 169-173.

15. Urhammer SA, Rasmussen SK, Kaisaki PJ, Oda N, Yamagata K, Moller AM *et al.*: **Genetic variation in the hepatocyte nuclear factor-1 alpha gene in Danish Caucasians with late-onset NIDDM.** *Diabetologia* 1997, **40:** 473-475.

16. Argyropoulos G, Rankinen T, Neufeld DR, Rice T, Province MA, Leon AS *et al.*: **A polymorphism in the human agouti-related protein is associated with late-onset obesity.** *J Clin Endocrinol Metab* 2002, **87:** 4198-4202.

17. Ogawa T, Hirose H, Miyashita K, Saito I, Saruta T: **GPR40 gene Arg211His polymorphism may contribute to the variation of insulin secretory capacity in Japanese men.** *Metabolism* 2005, **54:** 296-299.

18. Fenger M, Ambye L. Diagnosis and prognosis of metabolic diseases. PA 2003 00124. 2003. 31-1-2003.
Ref Type: Patent

19. Hansen SK, Rose CS, Glumer C, Drivsholm T, Borch-Johnsen K, Jorgensen T *et al.*: **Variation near the hepatocyte nuclear factor (HNF)-4alpha gene associates with type 2 diabetes in the Danish population.** *Diabetologia* 2005, **48:** 452-458.

20. Ek J, Rose CS, Jensen DP, Glumer C, Borch-Johnsen K, Jorgensen T *et al.*: **The functional Thr130Ile and Val255Met polymorphisms of the hepatocyte nuclear factor-4alpha (HNF4A): gene associations with type 2 diabetes or altered beta-cell function among Danes.** *J Clin Endocrinol Metab* 2005, **90:** 3054-3059.

21. Moller AM, Urhammer SA, Dalgaard LT, Reneland R, Berglund L, Hansen T *et al.*: **Studies of the genetic variability of the coding region of the hepatocyte nuclear factor-4alpha in Caucasians with maturity onset NIDDM.** *Diabetologia* 1997, **40:** 980-983.

22. Navas MA, Munoz-Elias EJ, Kim J, Shih D, Stoffel M: **Functional characterization of the MODY1 gene mutations HNF4(R127W), HNF4(V255M), and HNF4(E276Q).** *Diabetes* 1999, **48:** 1459-1465.

23. Lausen J, Thomas H, Lemm I, Bulman M, Borgschulze M, Lingott A *et al.*: **Naturally occurring mutations in the human HNF4alpha gene impair the function of the transcription factor to a varying degree.** *Nucleic Acids Res* 2000, **28:** 430-437.

24. Wermter AK, Reichwald K, Buch T, Geller F, Platzer C, Huse K *et al.*: **Mutation analysis of the MCHR1 gene in human obesity.** *Eur J Endocrinol* 2005, **152:** 851-862.
