# Supplementary material for: Analysis of heterogeneity and epistasis in physiological mixed populations by combined structural equation modelling and latent class analysis
Source: BMC Genet. 2008 Jul 8;9:43. doi: 10.1186/1471-2156-9-43 (PMC2483291; doi:10.1186/1471-2156-9-43)
Supplement: Additional File 2 — Comparisons of traits between the 19 classes. The table shows the number of significant differences of a trait between all two-class comparisons. [file 1471-2156-9-43-S2.pdf]

Table T2 Comparisons of traits between the 19 classes.

The table shows the number of significant differences of a trait between all two-class comparisons.

	Women				Men			
Significans level:	0.05		2.9 E-04[b]		0.05		2.9 E-04	
Trait	No. significant[a]		No. significant		No. significant		No. significant	
HOMAres	157	91.8%	137	80.1%	158	92.4%	146	85.4%
HOMAbeta	145	84.8%	112	65.5%	136	79.5%	102	59.6%
Insulin 0	157	91.8%	140	81.9%	157	91.8%	147	86.0%
Insulin 30	161	94.2%	153	89.5%	161	94.2%	153	89.5%
Insulin 120	162	94.7%	151	88.3%	163	95.3%	154	90.1%
C-peptid 0	151	88.3%	127	74.3%	155	90.6%	125	73.1%
C-peptid 30	165	96.5%	144	84.2%	158	92.4%	145	84.8%
C-peptid 120	160	93.6%	146	85.4%	155	90.6%	141	82.5%
Glucose 0	124	72.5%	52	30.4%	106	62.0%	51	29.8%
Glucose 30	121	70.8%	68	39.8%	101	59.1%	42	24.6%
Glucose 120	156	91.2%	128	74.9%	152	88.9%	128	74.9%
BMI	145	84.8%	98	57.3%	139	81.3%	95	55.6%
Cholesterol	95	55.6%	28	16.4%	76	44.4%	7	4.1%
HDL	122	71.3%	58	33.9%	120	70.2%	59	34.5%
LDL	75	43.9%	14	8.2%	42	24.6%	0	0.0%
VLDL	106	62.0%	29	17.0%	121	70.8%	44	25.7%
Triglycerides	125	73.1%	71	41.5%	117	68.4%	53	31.0%
WH	121	70.8%	64	37.4%	128	74.9%	69	40.4%
Systolic BP	107	62.6%	39	22.8%	97	56.7%	27	15.8%
Diastolic BP	109	63.7%	43	25.1%	106	62.0%	36	21.1%

[a] Total number of comparisons for a trait are 171

[b] Significance level after correcting for multiple testing (Bonferoni)
