# Supplementary material for: Analysis of heterogeneity and epistasis in physiological mixed populations by combined structural equation modelling and latent class analysis
Source: BMC Genet. 2008 Jul 8;9:43. doi: 10.1186/1471-2156-9-43 (PMC2483291; doi:10.1186/1471-2156-9-43)
Supplement: Additional File 3 — Summary of two-gene genetic variance as the fraction of total phenotypic variance in gender before stratification into subpopulations by SEM-LCA. The number of significant two-gene interactions and the variance decomposition for each variable examined in the study are summarized before stratification of each gender by SEM-LCA. [file 1471-2156-9-43-S3.pdf]

Table T3 Summary of two-gene genetic variance as the fraction of total phenotypic variance in gender before stratification into subpopulations by SEM-LCA
Variance components are averaged over all significant two-gene interactions

					Women						Men		
				Genetic variance[c]				Genetic variance					
Trait	Interactions[a]	Fraction[b]		Mean	Minimum	Maximum	Fraction	No interactions[a]	Fraction	Mean	Minimum	Maximum	Fraction
Age	183	42.1%	Total variance	2.74e-03	2.97e-04	9.37e-03		141	32.4%	2.64e-03	2.42e-04	1.08e-02	
Haplotypes[e]	15		Additive[d]	6.74e-04	7.52e-06	2.58e-03	24.6%	15		6.01e-04	4.01e-06	4.02e-03	22.8%
Real epistasis	168	38.6%	Dominance[d]	7.18e-04	2.98e-06	3.91e-03	26.2%	126	29.0%	5.19e-04	4.62e-06	2.90e-03	19.7%
			Epistasis	1.34e-03	5.00e-05	6.83e-03	49.1%			1.52e-03	2.73e-05	7.02e-03	57.5%
BMI	169	38.9%	Total variance	2.60e-03	3.10e-04	9.13e-03		149	34.3%	3.86e-03	3.88e-04	2.52e-02	
Haplotypes	13		Additive	6.41e-04	1.90e-06	1.90e-03	24.7%	14		1.12e-03	1.03e-05	9.22e-03	29.1%
Real epistasis	156	35.9%	Dominance	5.97e-04	8.00e-06	2.82e-03	23.0%	135	31.0%	1.02e-03	7.57e-06	1.28e-02	26.4%
			Epistasis	1.36e-03	3.27e-05	6.52e-03	52.4%			1.72e-03	1.80e-05	8.50e-03	44.4%
Cholesterol	179	41.1%	Total variance	3.65e-03	1.58e-04	1.76e-02		150	34.5%	4.53e-03	4.54e-04	1.21e-02	
Haplotypes	15		Additive	1.30e-03	1.29e-05	7.43e-03	35.6%	15		1.96e-03	3.92e-06	6.71e-03	43.2%
Real epistasis	164	37.7%	Dominance	8.94e-04	1.14e-06	7.28e-03	24.5%	135	31.0%	9.38e-04	2.02e-06	3.93e-03	20.7%
			Epistasis	1.46e-03	1.27e-05	7.65e-03	39.9%			1.64e-03	6.86e-05	9.10e-03	36.1%
C-peptide 0	165	37.9%	Total variance	2.26e-03	2.33e-04	1.01e-02		141	32.4%	2.57e-03	3.98e-04	1.06e-02	
Haplotypes	15		Additive	5.52e-04	1.90e-05	3.65e-03	24.4%	15		8.63e-04	1.63e-05	4.74e-03	33.6%
Real epistasis	150	34.5%	Dominance	5.20e-04	1.93e-06	3.63e-03	23.0%	126	29.0%	4.21e-04	1.77e-06	3.14e-03	16.4%
			Epistasis	1.19e-03	1.43e-05	7.10e-03	52.6%			1.29e-03	9.83e-06	6.61e-03	50.0%
C-peptide 30	178	40.9%	Total variance	2.90e-03	1.08e-04	1.46e-02		150	34.5%	3.15e-03	2.22e-04	1.22e-02	
Haplotypes	15		Additive	4.50e-04	7.60e-06	1.83e-03	15.5%	15		9.71e-04	8.36e-06	5.81e-03	30.8%
Real epistasis	163	37.5%	Dominance	7.87e-04	6.99e-07	3.77e-03	27.1%	135	31.0%	6.44e-04	4.13e-06	5.00e-03	20.4%
			Epistasis	1.67e-03	3.80e-06	1.14e-02	57.4%			1.54e-03	1.97e-05	7.91e-03	48.7%
C-peptide 120	177	40.7%	Total variance	2.86e-03	1.25e-04	2.56e-02		149	34.3%	3.04e-03	4.44e-04	1.06e-02	
Haplotypes	16		Additive	8.36e-04	2.09e-06	1.12e-02	29.2%	16		7.31e-04	1.90e-05	4.92e-03	24.0%
Real epistasis	161	37.0%	Dominance	5.30e-04	1.20e-05	4.21e-03	18.5%	133	30.6%	7.18e-04	1.82e-05	3.22e-03	23.6%
			Epistasis	1.50e-03	2.20e-05	1.23e-02	52.3%			1.60e-03	2.95e-05	6.26e-03	52.4%
Insulin 0	152	34.9%	Total variance	2.76e-03	2.13e-04	1.22e-02		137	31.5%	2.76e-03	5.33e-04	1.29e-02	
Haplotypes	17		Additive	9.83e-04	5.47e-06	6.61e-03	35.6%	15		1.10e-03	1.99e-06	5.11e-03	39.9%
Real epistasis	135	31.0%	Dominance	5.54e-04	1.47e-06	4.29e-03	20.1%	122	28.0%	4.07e-04	4.27e-07	2.46e-03	14.7%
			Epistasis	1.22e-03	1.92e-05	1.06e-02	44.3%			1.25e-03	2.78e-05	7.83e-03	45.4%
Insulin 30	177	40.7%	Total variance	2.68e-03	1.95e-04	1.45e-02		145	33.3%	3.78e-03	2.71e-04	2.44e-02	
Haplotypes	15		Additive	3.47e-04	6.10e-06	1.70e-03	12.9%	15		1.16e-03	2.10e-05	6.08e-03	30.8%
Real epistasis	162	37.2%	Dominance	8.02e-04	3.57e-07	3.54e-03	29.9%	130	29.9%	9.23e-04	3.66e-06	5.15e-03	24.4%
			Epistasis	1.53e-03	1.02e-05	1.32e-02	57.1%			1.69e-03	1.70e-05	1.75e-02	44.8%
Insulin 120	162	37.2%	Total variance	3.19e-03	3.66e-04	3.33e-02		134	30.8%	3.25e-03	3.21e-04	2.11e-02	
Haplotypes	15		Additive	1.04e-03	1.26e-05	1.02e-02	32.6%	15		8.28e-04	1.81e-05	5.63e-03	25.5%
Real epistasis	147	33.8%	Dominance	5.20e-04	9.67e-06	2.45e-03	16.3%	119	27.4%	8.22e-04	5.49e-06	4.63e-03	25.3%
			Epistasis	1.63e-03	1.86e-05	2.09e-02	51.1%			1.60e-03	5.32e-05	1.39e-02	49.3%
Glucose 0	146	33.6%	Total variance	2.44e-03	3.02e-04	2.17e-02		139	32.0%	4.72e-03	2.06e-04	3.77e-02	
Haplotypes	16		Additive	1.07e-03	7.74e-07	8.94e-03	44.0%	15		1.06e-03	1.55e-05	8.49e-03	22.6%
Real epistasis	130	29.9%	Dominance	4.24e-04	7.49e-07	3.43e-03	17.4%	124	28.5%	1.43e-03	2.33e-05	1.92e-02	30.4%
			Epistasis	9.42e-04	1.60e-06	9.29e-03	38.7%			2.22e-03	1.69e-05	1.81e-02	47.1%
Glucose 30	180	41.4%	Total variance	3.38e-03	3.55e-04	2.61e-02		161	37.0%	3.72e-03	6.16e-04	1.59e-02	
Haplotypes	18		Additive	1.59e-03	1.19e-05	1.68e-02	47.1%	14		9.27e-04	5.71e-06	4.60e-03	24.9%
Real epistasis	162	37.2%	Dominance	4.80e-04	6.53e-07	3.11e-03	14.2%	147	33.8%	8.62e-04	2.17e-06	7.87e-03	23.2%
			Epistasis	1.31e-03	3.12e-05	6.20e-03	38.7%			1.93e-03	6.48e-05	1.46e-02	51.9%
Glucose 120	174	40.0%	Total variance	3.21e-03	9.87e-05	2.78e-02		152	34.9%	3.22e-03	2.26e-04	1.02e-02	
Haplotypes	16		Additive	1.28e-03	1.42e-06	1.43e-02	40.0%	15		1.01e-03	1.10e-05	6.15e-03	31.5%
Real epistasis	158	36.3%	Dominance	4.95e-04	9.48e-06	3.98e-03	15.4%	137	31.5%	6.29e-04	6.97e-06	3.90e-03	19.5%
			Epistasis	1.43e-03	7.90e-06	1.27e-02	44.6%			1.58e-03	5.56e-05	6.72e-03	49.0%
H0MAres	123	28.3%	Total variance	2.14e-03	1.73e-04	8.95e-03		132	30.3%	2.84e-03	4.65e-04	1.54e-02	
Haplotypes	15		Additive	9.00e-04	1.58e-05	3.87e-03	42.1%	14		1.07e-03	6.32e-06	4.82e-03	37.6%
Real epistasis	108	24.8%	Dominance	4.02e-04	1.55e-06	2.51e-03	18.8%	118	27.1%	4.59e-04	1.09e-05	2.25e-03	16.2%
			Epistasis	8.38e-04	1.88e-05	6.59e-03	39.1%			1.31e-03	4.36e-05	1.31e-02	46.3%
HOMAbeta	156	35.9%	Total variance	3.61e-03	5.41e-05	8.10e-02		97	22.3%	2.81e-03	4.18e-04	1.30e-02	
Haplotypes	20		Additive	5.81e-04	8.35e-06	2.83e-03	16.1%	12		1.04e-03	8.84e-05	4.27e-03	36.9%
Real epistasis	136	31.3%	Dominance	5.86e-04	4.26e-06	4.94e-03	16.2%	85	19.5%	4.34e-04	7.48e-06	2.94e-03	15.5%
			Epistasis	2.44e-03	1.97e-05	7.59e-02	67.7%			1.34e-03	1.60e-05	8.19e-03	47.6%
Average													
Fractions		38.1%							32.5%				
Real epistasis		34.5%							29.1%				
Additive variance		30.3%							30.9%				
Dominance variance		20.8%							21.2%				
Total epistasis		48.9%							47.9%				

[a] Number of signficant two-gene epistatic effects corrected for multiple tests (cut-off 8,2E-06)

[b] Fractions of total number of possible epistatic effects (435 two-gene combinations)

[c] Variances expressed as the fraction of total phenotypic variance.

[d] The additive and dominant effects are for two-gene “haplotypes” (see text)

[e] Haplotypes denotes SNPs in physical linkage (maximum possible is 24)
