# Supplementary material for: Analysis of heterogeneity and epistasis in physiological mixed populations by combined structural equation modelling and latent class analysis
Source: BMC Genet. 2008 Jul 8;9:43. doi: 10.1186/1471-2156-9-43 (PMC2483291; doi:10.1186/1471-2156-9-43)
Supplement: Additional File 4 — Genes characterizing classes. The table summarize genes involved in epistasis of insulin, C-peptide or glucose in a limited number of classes. In particular, the MCHR1 gene or SNP is only involve in epistasis in one class in women and none in men. [file 1471-2156-9-43-S4.pdf]

Table T4 Genes characterizing classes[a]

Trait	Genes	Women Classes	Women Interactions	Men Classes	Men Interactions
Insulin 0	MCHR1	5	21	None[b]	
	AGRP	3, 10	34		
	HNF4α-5	3,5,10,11	56	5,8	11
	MTHFR	10	2		0
	GYS1	1,6,10	14		
	ARβ3			5,7,13,14	23
Insulin 30	MCHR1	5	21	None	
	AGRP	3,10,17	46		
	HNF4α-5	3,5,10,11	86	5,8,15,18	65
	MTHFR	4,5,6,9,10	26	6,8,9,15	19
	GYS1	1,2,3,6,10,16	92		
Insulin 120	MCHR1	5	23	None	
	AGRP	3,10,17	23		
	HNF4α-5	3,5,10,11	74	5,8,15,18	31
	MTHFR	4,5,6,9,10	16	9	2
	GYS1	1,2,3,6,10	66		
	ARβ3			4,5,7,13,14	37
C-peptide 0	MCHR1	5	21	None	
	AGRP	3,10,17	28		
	HNF4α-5	3,5,10,11	65	5,8,15,18	22
	MTHFR	10,11	2		0
	GYS1	1,2,3,6,10	43		
	ARβ3			5,7,12,13,14,19	37
C-peptide 30	MCHR1	5	23	None	
	AGRP	3,10,17	50		
	HNF4α-5	3,5,10,11	87	5,8,15,18	65
	MTHFR	4,5,6,9,10,11	30	6,8,9,15	19
	GYS1	1,2,3,6,10,16	92		
C-peptide 120	MCHR1	5	23	None	
	AGRP	3,10,17	45		
	HNF4α-5	3,5,10,11	82	5,8,15,18	55
	MTHFR	5,6,9,10,11	15	9,15,18	4
	GYS1	1,2,3,6,10,16	78		
	ARβ3			5,7,13,14,19	56
Glucose 0	MCHR1	5	24	None	
	AGRP	3,10,17	67		
	HNF4α-5	3,5,10,11	90	5,8,15,18	70
	MTHFR	12 classes	143		
	GYS1	1,2,3,6,10,16	127		
Glucose 30	MCHR1	5	20	None	
	AGRP	3,10,17	53		
	HNF4α-5	3,5,10,11	75	5,8,15,18	59
	MTHFR	4,5,6,10	14	8,9,13,15,16	8
	GYS1	1,2,3,6,10,16	88		
Glucose 120	MCHR1	5	24	None	
	AGRP	3,10,17	51	3,9,13,18	58
	HNF4α-5	3,5,10,11	83	5,8,15,18	57
	MTHFR	4,5,6,9,10	21	5,6,8,9,15	9
	GYS1	1,2,3,6,10,16	81		
	ARβ3			4,5,12,13	30

[a] Empty entries indicates that the gene is involved on two-gene interactions in more than 6 classes.

[b] MCHR1 does is not influence the traits in any two-gene interactions.
